# In Vitro and In Vivo Investigation of a Dual-Targeted Nanoemulsion Gel for the Amelioration of Psoriasis

**DOI:** 10.3390/gels9020112

**Published:** 2023-01-28

**Authors:** Rahmuddin Khan, Mohd. Aamir Mirza, Mohd Aqil, Thomson Santosh Alex, Nafis Raj, Nikhat Manzoor, Punnoth Poonkuzhi Naseef, Mohamed Saheer Kuruniyan, Zeenat Iqbal

**Affiliations:** 1 Department of Pharmaceutics, School of Pharmaceutical Education & Research (SPER), Jamia Hamdard, New Delhi 110062, India; 2 Department of Biosciences, Jamia Millia Islamia, New Delhi 110025, India; 3 Department of Pharmaceutics, Moulana College of Pharmacy, Perinthalmanna 679321, India; 4 Department of Dental Technology, College of Applied Medical Sciences, King Khalid University, Abha 61421, Saudi Arabia

**Keywords:** nanoemulsion gel, thymoquinone, imiquimod-induced psoriatic mice model, antioxidant, skin histopathology, immunohistochemistry

## Abstract

Psoriasis, due to its unique pathological manifestations and the limited success of existing therapeutic modalities, demands dedicated domain research. Our group has developed nanotherapeutics consisting of bioactives such as Thymoquinone (TQ) and Fulvic acid (FA), which have been successfully incorporated into a Nanoemulsion gel (NEG), taking kalonji oil as oil phase. The composition is aimed at ameliorating psoriasis with better therapeutic outcomes. TQ is a natural bio-active that has been linked to anti-psoriatic actions. FA has anti-inflammatory actions due to its free radical and oxidant-scavenging activity. Our previous publication reports the formulation development of the NEG, where we overcame the pharmaco-technical limitations of combining the above two natural bioactives. In vitro evaluation of the optimized NEG was carried out, which showed an enhanced dissolution rate and skin permeation of TQ. This work furthers the pharmaceutical progression of dual-targeted synergistic NEG to treat psoriasis. A suitable animal model, BALB/c mice, has been used to conduct the in vivo studies, which revealed the effective anti-psoriatic action of TQ. Molecular docking studies corroborated the results and revealed a good binding affinity for both the targets of TNF-α (Tumor necrosis factor) and IL-6 (Interlukin-6). Tissue uptake by Confocal laser scanning microscopy (CLSM), a skin interaction study of the gel formulation, and an antioxidant free radical scavenging assay (1-1 Diphenyl-2-picrylhydrazyl DPPH) were also carried out. It was concluded that the NEG may be effective in treating psoriasis with minimal side effects.

## 1. Introduction

Psoriasis is a chronic and an autoimmune inflammatory surface skin condition which affects 2–5% of the world’s population and is characterised by plaques and macules caused by hyperproliferation and improper keratinocyte differentiation [1,2,3]. The condition usually presents in late childhood or early adulthood (around the age of 40) and lasts a lifetime [3]. Psoriasis affects the skin as well as the joints, nails, tendons, bones, mucous membranes, and ligaments. The degree of psoriasis varies from red spots to complete body coverings and is clinically explained as the production of small restricted patches that span different bodily surface regions. The condition has a profound impact on the quality of life of patients who suffer from severe depression and suicidal ideation, resulting in high fatality rates [4]. Although the exact etiology of psoriasis is not yet elucidated, variables such as environment, heredity, immunological dysfunctions, and skin problems in relation to cutting, trauma, and stinging may impact its development [5,6] Available therapeutic modalities include topical, phototherapy, and systemic interventions which have fallen short in treating patients owing to a perquisition of a long therapeutic duration and consequent low patient compliance [7]. Psoriasis has also been linked to an increased risk of diabetes, cancer, and cardiovascular disease and often causes a reduced quality of life and exacerbates psychological stress [8]. Psoriasis patients’ rough and plaque-encrusted skin creates a considerable obstacle to topical medication delivery. Furthermore, the direct-indirect monetary value required to manage the disease influences patient therapeutic compliance [9]. Present medications for psoriasis effectively address symptoms, but have no effect on the underlying root cause of the disease and show various negative side effects [10]. Despite these hurdles, topical therapy remains the standard-of-care approach for psoriasis and is used alone in mild instances or in combination with phototherapy, biological agents, and systemic therapy in more critical cases [11].

TQ is a lipid-soluble benzoquinone that is a key active component of Nigella sativa, [12] which is an extensively used plant in traditional medicine with therapeutic benefits for several ailments and disorders [13]. Clinical studies are available to showcase the utility of Nigella sativa for the treatment of psoriasis, where 65% of patients found it safe and therapeutically successful, while approximately 31% showed relapse after discontinuing the therapy for 4 weeks [14,15]. TQ also acts as a scavenger of free radicals and superoxide radicals and maintains the activity of antioxidant enzymes such as glutathione peroxidase, catalase, and glutathione-S-transferase [10]. In psoriatic lesions, TQ suppresses epidermal proliferation and differentiation via inhibiting dendritic cells and the generation of IL-6 and TNF-α by epidermal T cells [16,17]. TQ is limited by its poor absorption and inadequate water solubility, leading to its poor oral therapeutic potential.

FA falls under the category of humic substances and has antibacterial, anti-inflammatory, and antifungal effects of humic substances and is a subclass of other chemicals that are byproducts of organic breakdown by bacteria. In addition, it has recently been revealed that FA is a non-toxic and anti-inflammatory agent when employed in the treatment of rat wound model [18]. The capacity of FA to scavenge oxidants and decrease their production reiterates its antioxidants actions [19,20,21,22]. Several investigations have found that FA can reduce the release of pro-inflammatory mediators from cells, making it an anti-inflammatory agent [17,23,24]. When given topically to healthy volunteers, it did not influence safety metrics and was unable to cause any sensitization [25].

In the current investigation, we have successfully developed an NEG for the effective and safe therapy of psoriasis. The NEG offers dermal safety and high anti-psoriatic action. The hypothesis behind the research was that a set of two medications, one with anti-psoriatic activity and another with anti-inflammatory actions, can be used in combination to treat psoriasis [26]. Owing to their local action and no or reduced systemic adverse effects, topical administration techniques have significant advantages. Nanoemulsions as topical delivery vehicles are viable since they are transparent and kinetically stable systems with a particle size between 20 and 200 nm [27]. Owing to its lipidic composition and tiny globule size, nanoemulsion (NE) is thought to penetrate psoriatic skin’s tough plaques by extracting and expanding skin lipids, allowing for better penetration via the pores [28]. NEG furthers the delivery of drugs into the skin through a hydration mechanism, leading to an improvement in drug retention [29,30]. Combination drug therapy has different pharmacodynamic actions in comparison to sole agents. The rationale behind formulating the herbal drug NE is to attain synergistic actions, reduced side effects, and have increased therapeutic actions.

For the given project, NEG was developed and characterised in vitro in our previous publication [31]. In short, the NE was formulated using an aqueous titration method. In this method, kalonji oil was taken as oil phase while Tween 80 and Transcutol-P were selected as the surfactant and co-surfactant, respectively. A pseudo-ternary phase diagram was plotted for the selection of the final NE composition. The BBD optimization method was applied to obtain the optimized formula. The optimized composition was as follows: characteristics—composition (oil 1.75%, S^mix^(2:1) 12%, water 95%) The particle size, PDI, and % transmittance were found to be 72.34 ± 2.43 nm, 0.126 ± 0.014, and 98.99 ± 0.22%, respectively. The NEG also showed a sustained dissolution rate and permeation (75.76 ± 3.62%, 3.64 μg/cm^2^/h). 

In this manuscript, the previously reported formulation [31] was tested using an antioxidant free radical scavenging assay (DPPH), CLSM, a skin interaction study, and in vivo anti-psoriatic activity. The hypothesis was also tested using in silico molecular docking studies.

## 2. Results and Discussion

### 2.1. In Silico Molecular Docking Study

**Figure 1:**
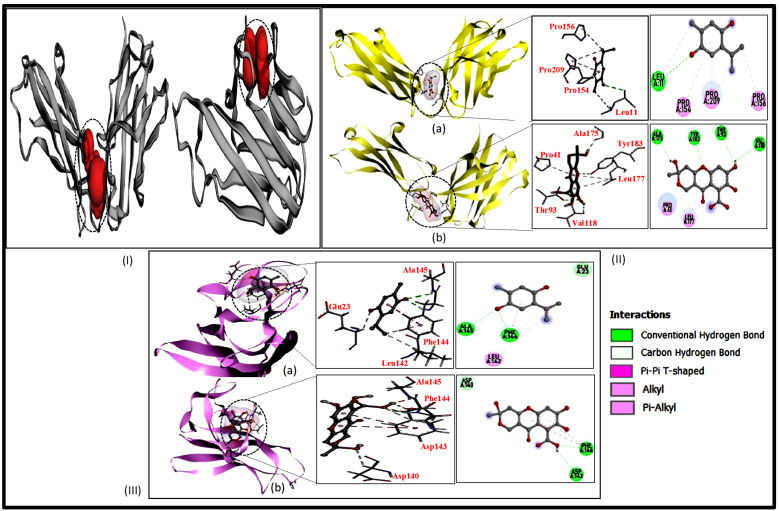
(**I**) The binding pocket (red cartoon with dotted line) of 4CNI and 6RMJ. (**II**) The 3D interaction of IL-6 (4CNI) with TQ (a) and FA (b). (**III**) Cartoon representation showing the hydrogen bond interaction of TNF (6RMJ) with TQ (a) and FA (b).

Molecular docking studies showed that the docking scores of TQ and FA were −4.4 and −6.0 kcal/mol, respectively, with IL-6 and the residues Leu11, Val118, Thr93, Ala175, and Tyr183 forming polar hydrogen bonds with the ligands as well as hydrophobic bond interactions (van der walls, alkyl, pi–alkyl, pi–pi, T-shaped) with Pro54, Pro56, Pro209, Leu177, and Pro41 (Table 1). However, the docking score of both compounds with TNF-α were −4.8 and −6.4 kcal/mol, respectively, and interacting residues Ala145, Phe144, Asp143, Phe144, and Asp140 showed the polar bonds and hydrophobic interactions with Phe144, Leu142 (interaction shown in Figure 1 (I, II and III)). After the molecular docking study by Auto dock vina tools was conducted, the results indicated that the FA have a significant binding score (−6.0 and 6.4 kcal/mol) and a high affinity with both receptors. Molecular docking has recently emerged as a dependable tool to envisage the actual behaviour of the drug at the site of action and henceforth warrants better therapeutic outcomes [32].

### 2.2. In Vitro Studies

#### 2.2.1. Antioxidant DPPH Free Radical Scavenging Assay

**Figure 2:**
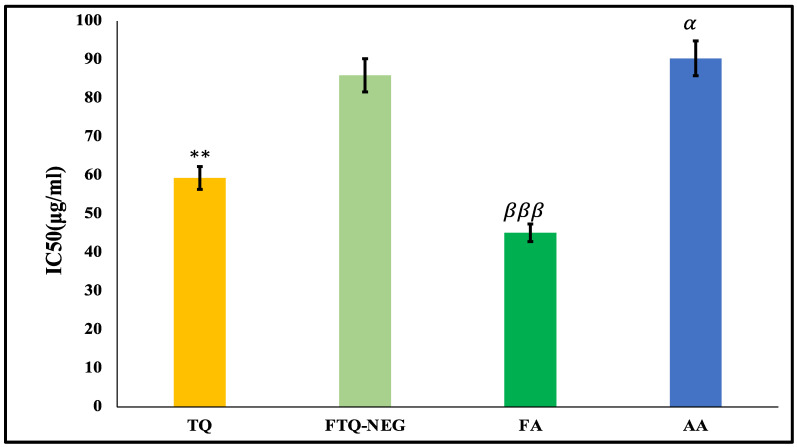
Free radical scavenging activity of the study groups; all the data were found to be significant. Data are expressed as mean ± SD (** *p* < 0.01, FTQ-NEG vs. TQ), (βββ *p* < 0.001, FTQ-NEG vs. FA), (α *p* < 0.05, FTQ-NEG vs. AA).

The quantity necessary to reduce the primary concentration of DPPH by 50%, also called the IC50, indicates the anti-oxidant potential of any substance. The findings indicated the extent of discoloration and substantial scavenging potential by the free radicals and compared the radical scavenging capacity by DPPH of TQ, FTQ-NEG, FA, and ascorbic acid (AA) (standard). The following IC50 values were obtained: TQ (59.32 ± 1.21 μg/mL); FTQ-NEG (85.93 ± 1.3 μg/mL); AA (90.34 ± 2.03 μg/mL); and FA (45.12 ± 1.1 μg/mL). The data were found to be significant as shown in Figure 2 [33].

#### 2.2.2. Skin Interaction Study of the Gel Formulation 

**Figure 3:**
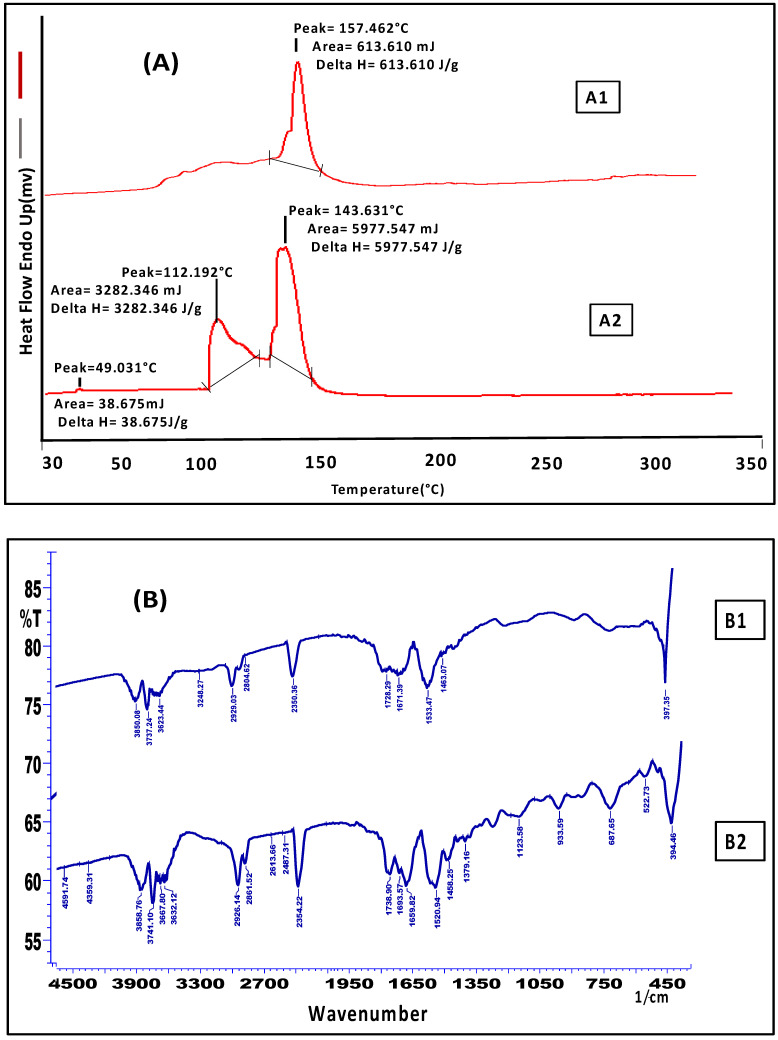
(**A**) DSC thermogram of mice skin. (A1) Untreated, (A2) Treated with optimized FTQ-NEG. (**B**) FTIR spectra of mice skin. (B1) Untreated, (B2) Treated with optimized FTQ-NEG.

In the skin interaction tests, the preparation- (FTQ-NEG) treated skin of mice displayed endotherms (Tm), and DSC thermogram enthalpy (∆H) matched with untreated skin of mice. The skin of untreated mice exhibited a ∆H and Tm of 5977.5 J/g and 143.6 °C, respectively (Figure 3(A1)). The ∆H levels were lower in the treated skin. In contrast with the control/untreated skin, the Tm value of the treated skin was 157.4 °C, whereas ∆H sharply decreased to 613.6 J/g (Figure 3(A2)). The reduction in ∆H esteem leads to skin lipid bilayer disruption, and an increment in Tm esteem leads to the fluidization of the lipid bilayer [34]. The stratum corneum (SC) protein denaturation may have caused an extra peak at 112.1 °C in the untreated skin. FTIR spectra of untreated skin exhibited distinct peaks owing to the molecular vibration of proteins and lipids present in the SC due to the fluidization of loosely linked SC lipids and proteins by the surfactant in the formulation (Figure 3(B1)). The C-H stretching of the alkyl groups in both proteins and lipids were responsible for these absorption bonds. For the spectra of mice skin, the proteins of the stratum corneum were specified bands. After treatment with FTQ-NEG, the stretching vibrations of amide I (1658–1740 cm^−1^), amide II (1520–1533 cm^−1^), and amide III (1458–1463 cm^−1^) changed (Figure 3(B2)). This can be attributable to the formulation’s small interaction with skin proteins. 

#### 2.2.3. CLSM Study for Tissue Uptake

**Figure 4:**
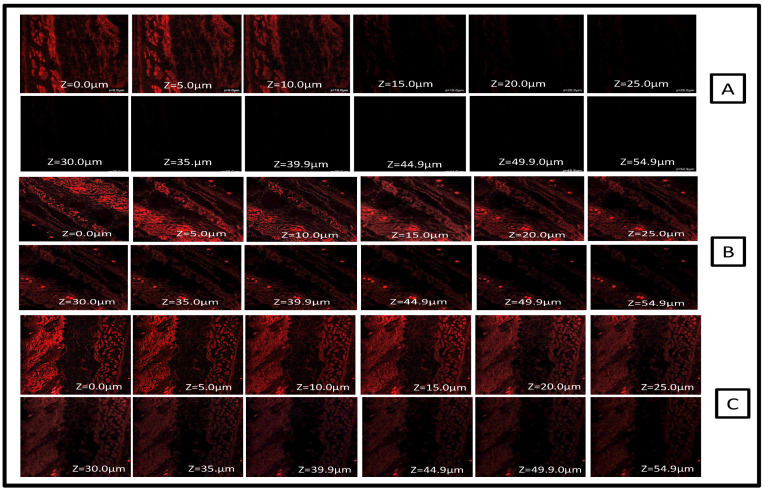
Confocal laser scanning microscopy in optical sectioning of tissues. (**A**) pure drug TQ (**B**) FTQ-NEG normal skin (**C**) FTQ-NEG with psoriatic skin.

The optical visualization of tissues was completed using depth-dependent distribution of the fluorescent probe without any need of sectioning and tissue fixation; indeed, this is the topmost advantage of CLSM. The CLSM technique has been utilized for depicting the drug skin penetration mechanism using aqueous dispersion of Rhodamine B dye. When comparing the FTQ-NEG aqueous dispersion with the pure drug solution, the drug solution had meagre fluorescent strength up to 15 μm (Figure 4A). This might be due to the aqueous dye solution causing less interference and staying in the surface layers of the skin; the solution did not penetrate deeply, but in the FTQ-NEG penetration was up to 54.9 μm (Figure 4B) and revealed a profound luminous intensity in the viable dermis and epidermis. The psoriatic skin absorbed rhodamine B-loaded FTQ-NEG, and a probe penetration up to 54.9 μm (Figure 4C) was also observed after 24 h and the delivery of sufficient Rhodamine B. FA and Tween 80 are the established permeation enhancers which were used in formulating NEG; they aid in crossing the stratum corneum (SC), which is a barrier to medications passing through the scaly psoriatic skin layer [35].

### 2.3. In Vivo Animal Study 

#### 2.3.1. Skin Irritation Study

**Figure 5:**
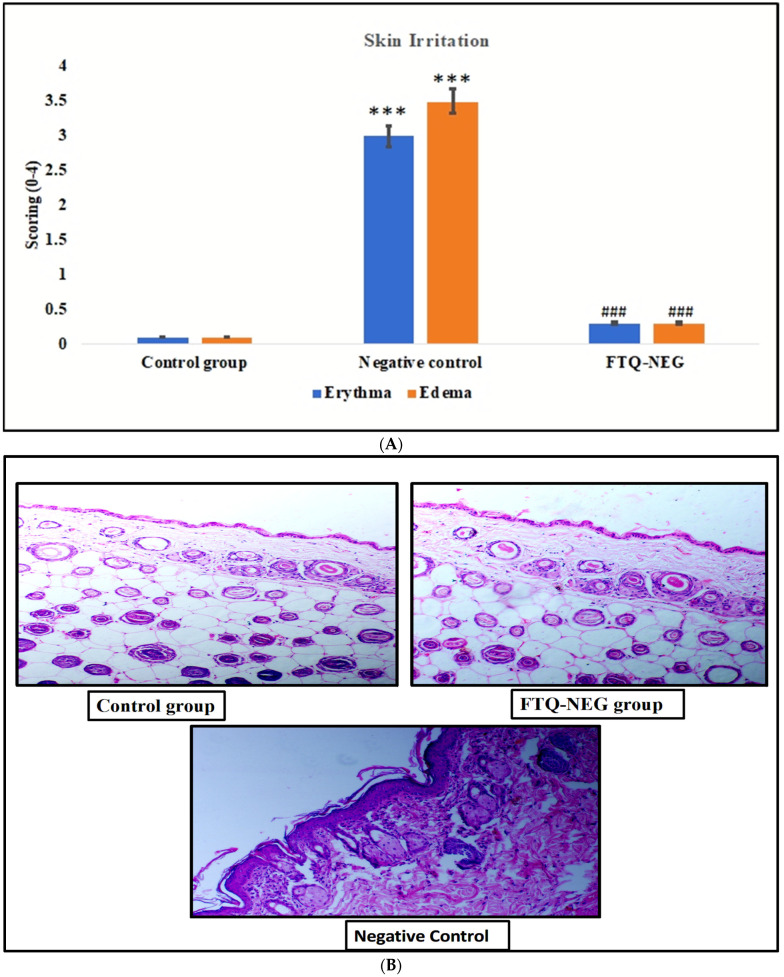
(**A**) The skin irritation potential score of all the groups. Data are expressed as mean ± SD (*** *p* < 0.001, control group vs negative control for erythema and edema) and (^###^ *p* < 0.001, negative control vs FTQ-NEG for erythema and edema). (**B**) Histopathology of all the treatment groups: Control, FTQ-NEG, and Negative control.

The visual grading system was used to provide a cutaneous irritation score as shown in (Figure 5A). After administering the dermal gel formulation, the FTQ-NEG-formulation-treated group showed neither erythema nor edema on the skin, with average scores of (0.3 ± 0.02) and (0.3 ± 0.03), respectively, and no remarkable changes in the skin condition were recorded. Higher erythema (3 ± 0.32) was noted in the Negative group treated with the standard irritant formalin solution, and a higher edema (3.5 ± 0.33) irritation score than in the Control group (0.1 ± 0.01) and (0.1 ± 0.01). The primary irritation index (PII) was calculated using the erythema and edema scores, and the following result was observed for the FTQ-NEG, Negative control, and Control group: PII: −0.6, 6.5, and 0.2, respectively. Scores of 2 or less are rated as negative. The findings of cutaneous irritation show that they are non-irritants, with a score of less than 2. As a result, the dermal FTQ-NEG formulations created are devoid of skin irritation. Histopathological finding has been conducted for the further confirmation of the internal changes; we found that the control group and FTQ-NEG formulation group have similar internal skin structure, but the group treated with the standard irritant formalin experienced a change in the internal skin and have erythema and edema (Figure 5B) [36].

#### 2.3.2. Imiquimod- (IMQ) Induced Psoriatic Plaque-Like Model

**Figure 6:**
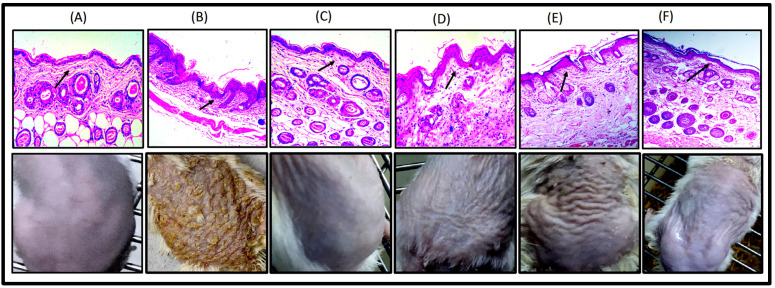
Antipsoriatic experiment in vivo results. Sample picture of balb/c mice from each experimental group: (**A**) Control group, (**B**) Negative control, (**C**) FTQ-NEG, (**D**) Positive control group, (**E**) Free drug, and (**F**) FA loaded in a gel, along with H&E images of histopathological sections from all research groups. Psoriatic plaques in the histological sections are represented with the black arrows.

Within 3–5 days after starting IMQ therapy, the dorsal skin of the IMQ-treated mice started to show symptoms of thickness, moderate scaling, and erythema. There was noticeable inflammation that progressively worsened by days 6–8. After the development of the animal model, a treatment gel was applied once per day. There was a reduction in all visual indications such as swelling and redness in the FTQ-NEG-treated group. All the parameters in the control group remained unchanged. The score of scaling, erythema, and thickness decreased starting on the second day, and on the sixth day, the score was zero; this was consistent with respect to control group. All these measures declined in the group treated with the positive control (Clobetasol propionate gel) and the free drug, but to a lower extent than the FTQ-NEG-treated group. The FTQ-NEG-treated group had significantly lower cumulative scores than the standard-treated group (Clobetasol propionate gel). Based on the results obtained from the scoring of all groups, we can infer that the developed formulation (FTQ-NEG) was more promising in terms of efficacy than the standard (Clobetasol propionate gel) formulation. For all eight days, the Negative control group had a notable high score. A histopathological investigation was also performed on all the groups of skin to look for any internal changes, as shown in (Figure 6A). In the case of the control skin and tissue lined with keratinized stratified squamous epithelium, intact epidermis and viable dermis were produced. Normal skin appendages (sebaceous glands and hair follicles) and a modest inflammatory cell infiltration can be seen in the sub-epithelium (Figure 6C). Skin treated with the developed formulation FTQ-NEG revealed no changes in skin architecture, and keratinized stratified squamous epithelium lining the minor inflammatory cell infiltration and normal skin appendages can also be seen in the sub-epithelium (sebaceous glands and hair follicles). The thickness and appearance of the horny layer were determined and found to be unaffected (Figure 6B). The epidermis and dermis of the Negative-control-treated skin showed visible alterations, as well as an aberrant internal skin organisation. Acanthosis and horn cysts arise in tissue bordered by keratinized stratified squamous epithelium (Figure 6E). Sub-epithelium reveals considerable inflammatory cell infiltration and enlarged skin appendages in the case of free drugs (Figure 6D). In the case of the positive control, there is a slight inflammatory cell infiltration as well as a reduction in skin appendages (Figure 6F). Sub-epithelium shows a mild inflammatory cell infiltrate and normal skin appendages [37]. 

#### 2.3.3. Ear Thickness and Inflammation Measurement via PASI (Psoriasis Area Severity Index) Scoring

**Figure 7:**
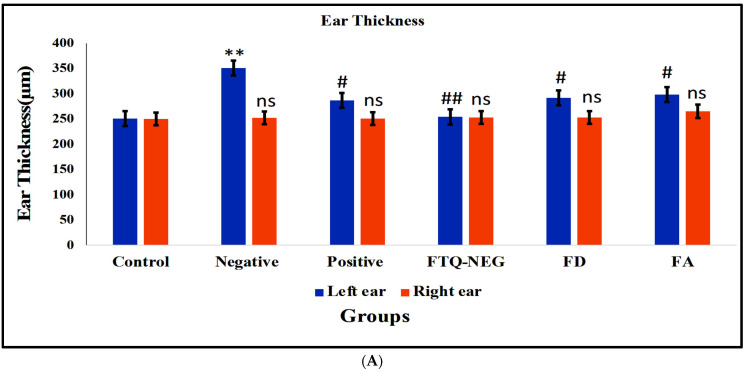

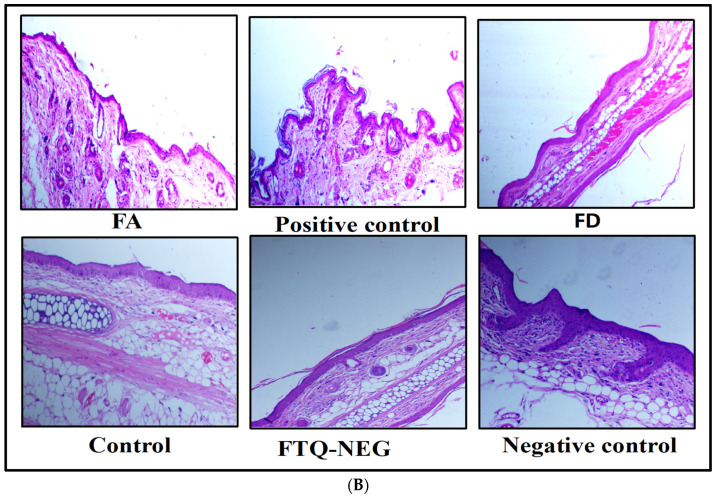
(**A**) Evaluation of ear thickness of all the groups. Data are expressed as mean ± SD. Ear thickness was increased in the negative control group in the left ear (** *p* < 0.01, control group vs negative control for left ear thickness) and decreased in the positive control, FTQ-NEG, FDG, and FA groups compare to the negative control (^#^ *p* < 0.05), ^##^ *p* < 0.01 vs negative control) in the left ear and non-significant (^ns^ *p* > 0.05 vs control) in the right ear. (**B**) histopathology of the left and right ear of mouse skin of all the groups: Control, Negative control, Positive control, FTQ-NEG, FD, and FA.

Ear thickness measurements are shown in Figure 7A. The negative group had a left ear thickness of >350 μm, whereas the control group had a right ear thickness of 251 μm. Left ear thickness decreased significantly in all treatment groups; however, it was lowest in the FTQ-NEG group. The histological hematoxylin- and eosin-stained sections of the left ear indicate the results obtained with the PASI score and ear thickness measurement, showing increased epidermal thickness and keratinocyte infiltration with rete ridges in the negative control group, but intact skin with no sign of thickness and infiltration in the Control group, similar to the FTQ-NEG group. The positive control group (marketed group), FD group, and FA group showed an alteration of the internal structure of the ear (Figure 7B). Except for the negative control (disease caused group), in both of the therapy groups, there was no significant change in left or right ear thickness, but there was significant inflammation and thickening [38].

##### Bodyweight and PASI Scoring 

**Figure 8:**
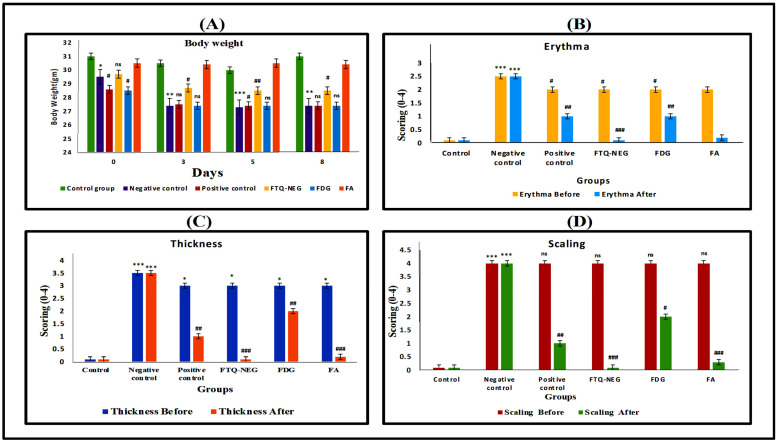
Efficacy study of all the groups. Data are presented as mean ± SD. (**A**) Body weight was decreased in negative control group when compared to control group (* *p* < 0.05, control vs negative control on 0th day), (** *p* < 0.01, control vs negative control on 3rd day & 8th day), (*** *p* < 0.001, on 5th day) but increased in positive group in comparison to negative control (^#^ *p* < 0.05). (**B**) Erythema scoring was significantly increased in negative control group when compared to control group (*** *p* < 0.001) and decreased in positive control, FTQ-NEG, and FDG groups compare to negative control (^#^ *p* < 0.05, ^##^ *p* < 0.01), (^###^ *p* < 0.001) in erythema before and after. (**C**) In thickness scoring, negative control group was significantly increased compared to control group (*** *p* < 0.001) and decreased in positive control, FTQ-NEG, and FDG groups compared to negative control (^##^ *p* < 0.01) in thickness before and after. (**D**) In scaling scoring, the negative control group was significantly increased compared to control group (*** *p* < 0.001) and decreased in positive control, FTQ-NEG, and FDG groups compared to negative control (^##^ *p* < 0.01) in scaling score after and non-significant in scaling score before.

During the eight-day treatment period, the body weights of all the groups were measured. Until the third day of the trial, all groups except the control group had little decrease in body weight; after that, there was no change in body weight as described in (Figure 8A). The PASI score is one of the metrics used to assess the effectiveness of a product. It is plotted on a scale of 0–4 on day zero, three, five, and eight. The degree of vasodilation caused by the production of cytokines (TNF-α and IL-6) and various substances such as NO, histamine, phospholipase A2 metabolites, and other compounds in the dermis by dendritic cells, mast cells, keratinocytes, and other cells is referred to as erythema. Pro-inflammatory cytokines cause keratinocytes to proliferate, resulting in increased skin thickness. Scaling arises due to the lack of differentiation in keratinocytes. On the eighth day of IMQ treatment, the negative control (disease-induced group) showed scaling, thickness, and erythema with a marking of 3–4, which falls in the severe category, indicating that inflammatory responses have formed, in contrast with the control group [39]. In opposition to the negative control group, the positive control group (Marketed formulation), FD in gel and FTQ-NEG exhibited a substantial reduction in erythema, skin thickness, and scaling (Figure 8B–D).

#### 2.3.4. Spleen Weight and Histopathology

**Figure 9:**
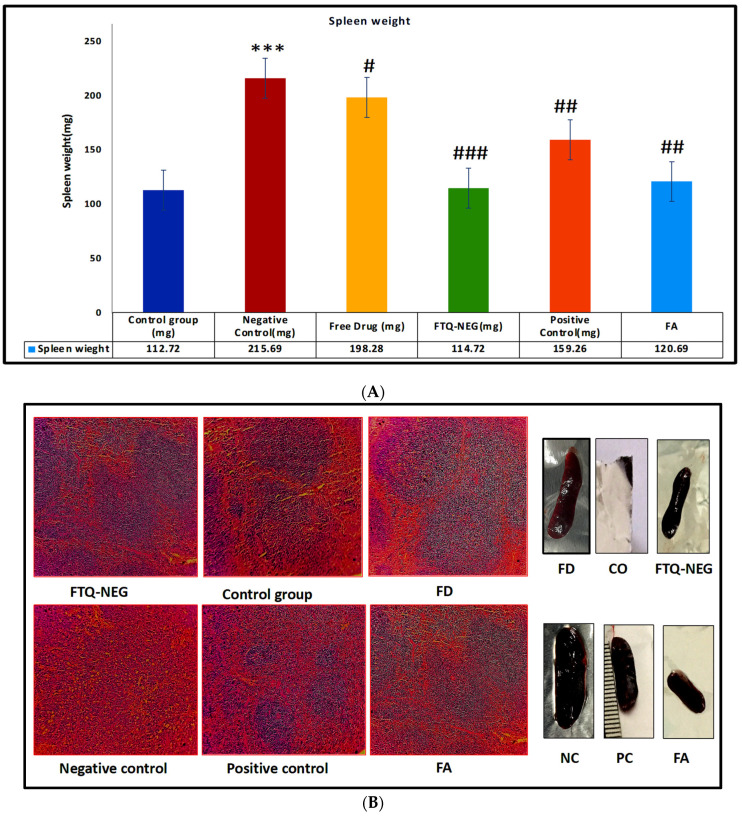
(**A**) Data are presented as mean ± SD. Spleen weight decreased in negative control group when compared to control group (*** *p* < 0.001) but increased in free drug, FTQ-NEG, positive control, and FA groups in comparison to negative control (^#^ *p* < 0.05, negative control vs free drug), (^##^ *p* < 0.01, positive control & FA vs negative control),( ^###^ *p* < 0.001, FTQ-NEG vs negative control). (**B**) Histopathology of the spleen tissue of all the groups: Control, Negative control, FD, FTQ-NEG, Positive control, and FA.

On the eighth day of the trial, the spleen weight of each study group was determined. The negative control group gained weight, indicating that psoriasis was induced following the administration of IMQ. Other treatment groups also saw a decline, but there was no noticeable difference between them. Figure 9A depicts the results of spleen dimensions. The negative group had an average spleen weight of 215.69 ± 13.20 mg, while the positive group had an average of 159.26 ± 12.34 mg. In addition, the FD in the gel was 198.28 ± 14.27 mg. The average weight of the spleen in the FTQ-NEG group was 114.12 ± 5.67 mg, which was similar to the average weight of the control group, 112.72 ± 7.23 mg. The spleens of the negative control group were largest, followed by the positive control group spleens in a decreasing order. The histology of the spleen (Figure 9B) of the FTQ-NEG group revealed full remission of spleen tissues, with blue indicating the return of white pulp and pink indicating red pulp sections of the spleen, as in typical spleen tissue [40]. Primary lymphoid follicles of various sizes are seen in the white pulp. Dilated sinusoids containing RBCs can be seen in the red pulp. The red and white pulp portions are not distinguishable in the negative control group. These lymphoid follicles consolidate and encroach on the red pulp, engulfing the bulk of the red pulp. White pulp from both the FD and positive control group demonstrates the establishment of primary lymphoid follicles that are smaller in size. In FA and FD, similar but minor splenic tissue repair was observed [40].

#### 2.3.5. Immunohistochemistry Staining (IHC) of TNF-α and IL-6 in Skin and Spleen Tissue

##### Skin Tissue

**Figure 10:**
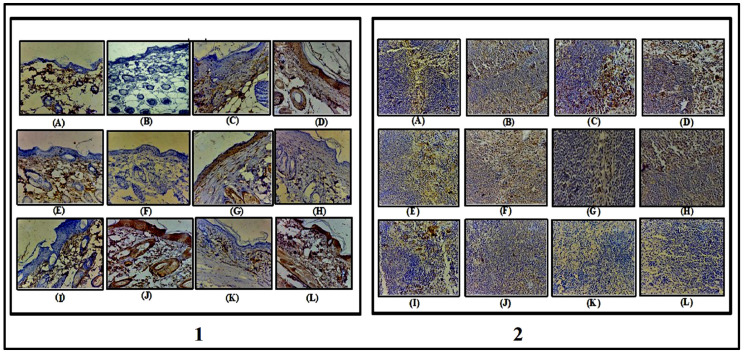
Immunohistochemistry of skin (**1**): (A,B) Control group of TNF-α and IL-6, (C,D) Negative control, (E,F) FTQ-NEG, (G,H) FA, (I,J) FD gel, (K,L) Positive control. (**2**) Immunohistochemistry of spleen: (A,B) Control group of TNF-α and IL-6, (C,D) Negative control group, (E,F) FTQ-NEG group, (G,H) FA, (I,J) FD gel, (K,L) Positive control (IHC, TNF-α, IL-6 400×).

The section examined (Figure 10(1)) shown in (Figure 10A,B) of a control group of TNF-α and IL-6 in skin shows negative expression in epidermal cells while scattered cells in the upper dermis show mild cytoplasmic positivity for TNF-α. Skin adnexal structures (hair follicles and sebaceous glands) also show negative expression (IHC, TNF-α, 400×), and skin shows mild cytoplasmic positivity expression of IL-6 in epidermal cells and in few cells of the upper dermis. Scattered hair follicles and sebaceous glands also show positive expression (IHC, IL 6, 400×). In the case of (Figure 10C,D), the Negative control group shows negative expression in epidermal cells while scattered cells in the upper dermis show mild cytoplasmic positivity for TNF-α. Skin adnexal structures (hair follicles and sebaceous glands) also show negative expression (IHC, TNF-α, and 400×), cytoplasmic positivity expression of IL-6 in epidermal cells, and focal positivity in fibrotic area of upper dermis. Scattered hair follicles and sebaceous glands also show positive expression (Figure 10E,F). Skin shows negative expression in epidermal cells, while scattered cells in the upper dermis show mild cytoplasmic positivity for TNF-α. Skin adnexal structures (hair follicles and sebaceous glands) also show negative expression (IHC, TNF-α, 400×), and skin shows the negative expression of IL-6 in epidermal cells, dermis, and skin adnexal structures (hair follicles and sebaceous glands) (IHC IL-6, 400×). (Figure 10G,H) skin shows negative expression of TNF-α in epidermal cells, dermis, and in skin adnexal structures (hair follicles and sebaceous glands) (IHC TNF-α, 400×). Skin shows mild cytoplasmic positivity expression of IL-6 in epidermal cells and focal positivity in fibrotic areas of the upper dermis. Scattered hair follicles and sebaceous glands also show positive expression (IHC, IL 6, and 400×) (Figure 10I,J). Skin shows negative expression in epidermal cells, while scattered cells in the upper dermis show mild cytoplasmic positivity for TNF-α. Skin adnexal structures (hair follicles and sebaceous glands) also show negative expression (IHC, TNF-α, and 400×). Skin shows mild cytoplasmic positivity expression of IL-6 in epidermal cells and a few cells of upper dermis. Scattered hair follicles and sebaceous glands also shows positive expression (IHC, IL-6, and 400×). (Figure 10K,L) skin shows negative expression in epidermal cells, while scattered cells in the upper dermis show mild cytoplasmic positivity for TNF-α. Skin adnexal structures (hair follicles and sebaceous glands) also show negative expression (IHC, TNF-α, and 400×). From skin shows mild cytoplasmic positivity expression of IL-6 in epidermal cells and a few cells of the upper dermis. Scattered hair follicles and sebaceous glands also show positive expression (IHC, IL-6, 400×) [41].

##### Spleen Tissue

Figure 10(2) (A,B) shows, in the control group, TNF-α and IL-6 mild cytoplasmic positivity predominantly in red pulp and negative expression in white pulp (IHC, TNF-α, 400×). The section examined shows mild cytoplasmic positivity predominantly in red pulp and the follicle of white pulp (IHC, IL-6, 400×). (Figure 10C,D) disease-induced group shows mild cytoplasmic positivity of TNF-α in red pulp and in scattered cells of lymphoid follicles of white pulp (IHC TNF-α, 400×), examined shows weak cytoplasmic positivity of IL-6 in lymphoid follicles and scattered cells of red pulp in spleen (IH, IL-6, 400×). FTQ-NEG examined in (Figure 10E,F) shows mild cytoplasmic positivity predominantly in red pulp and also in few scattered cells of the follicle center in white pulp (IHC, TNF-α, 400×), and examined from spleen shows mild cytoplasmic positivity seen in follicles of white pulp and scattered cells in the red pulp area (IHC, IL-6, 400×). In (Figure 10G,H), mild cytoplasmic positivity is shown predominantly in red pulp and negative expression in white pulp (IHC, TNF-α, 400×), with mild cytoplasmic positivity predominantly in red pulp and in the follicles of white pulp (IHC, IL-6, 400×). Figure 10I,J shows mild cytoplasmic positivity predominantly in red pulp and also in few scattered cells of the follicle center in white pulp (IHC, TNF-α, 400X), and in spleen shows mild cytoplasmic positivity in follicles of white pulp and scattered cells in the red pulp area (IHC, IL-6, 400×). (Figure 10K,L) shows the negative expression of IHC TNF-α in white pulp and red pulp of the spleen (IHC TNF-α, 400×); the section examined shows the negative expression of IHC IL-6 in white pulp and red pulp of the spleen (IHC IL-6, 400×) [42].

#### 2.3.6. Analysis of Cytokine Levels in Skin Homogenate

**Figure 11:**
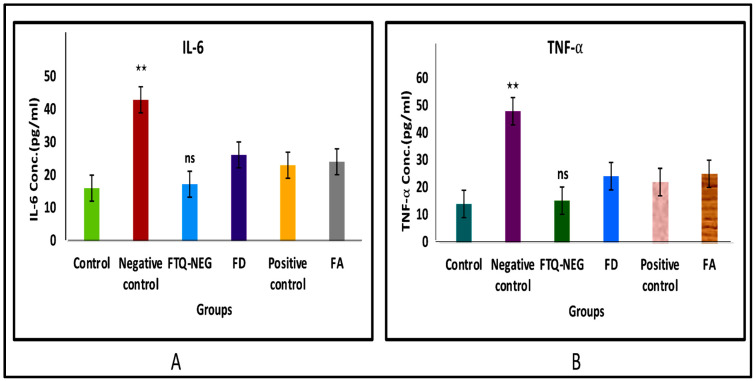
Levels of IL-6 (**A**) and TNF-α (**B**) that were assessed in skin homogenate of different animal groups: Control, Negative control, FTQ-NEG, FD, Positive control, and FA (** indicate *p* < 0.01) and ns is non-significant).

The effectiveness of topically applied FTQ-NEG was assessed in this study by measuring the concentration of IL-6 and TNF-α cytokines in the skin homogenates taken on the eighth day of the work, as the discussed cytokines are vital in psoriasis. Figure 11 shows the amounts of TNF-α and IL-6 identified in skin homogenates of animals from various groups in contrast to the control group. The negative control group had a considerable rise in TNF-α and IL-6 levels, demonstrating successful model development. TNF-α levels in the FTQ-NEG group were lower than in the negative control group. Similarly, IL-6 concentrations were also reduced in the FTQ-NEG treatment group, in contrast with the negative control group, and similarly in other groups [43].

## 3. Conclusions

This project proposes a combination therapy of TQ and FA, utilizing kalonji as the oil phase and Tween 80 as a surfactant, which offers effective penetration through the skin. Furthermore, the test conducted for skin irritation was directed towards the safety profile of FTQ-NEG via a topical route, showing that it has no irritant potential, indicating the potential of long-term therapy. The findings are encouraging, proving that FTQ-NEG is safe and efficacious and demonstrating a viable method for developing topical psoriasis treatments. Topical application of the FTQ-NEG showed improvement in the symptoms of psoriasis in the IMQ-induced psoriatic plaque model. The findings of PASI scoring, histological investigation, and ELISA were compared and revealed that FTQ-NEG can ameliorate and cure psoriasis. To summarise, FTQ-NEG was shown to be more successful in treating psoriasis than commercially available treatments. The new formulation increased the site concentration of both medications, lowering the systemic adverse effects and allowing for more penetration into the epidermal layers, which was previously impossible with standard formulations. In the current study, the combined treatment method promotes psoriasis recovery. When compared to the gel-containing free drug, the current method causes less skin irritation. In addition, compared to the other trial groups, the local topical concentration improved. This method provides a customised and acceptable safety profile. Long-term therapy is likely to be achievable with good patient compliance thanks to the formulation’s lower irritation potential. This encourages the future applicability and commercialization of the formula.

## 4. Materials and Methods

### 4.1. Material

The FA was received as a gift sample from NZ Fulvic Ltd., New Zealand. TQ, DPPH, and Rhodamine B were purchased from Sigma-Aldrich (Darmstadt, Germany). Transcutol P, Capryol 90, Labrasol, Plurol, Polyethylene glycol, Tween 20, Tween 80, and PEG 200 were obtained from Gattefosse Pvt. Ltd., Mumbai, India as gift samples. TNF-α and IL-6 ELISA Kits were purchased from Krishgen Biosystem (Mumbai, India). Imiquimod (IMQ) 5% cream (Glenmark Pharmaceuticals Ltd. Imiquad^®^, (Delhi, India) was purchased from a local pharmacy. Castor oil, Kalonji oil, lavender oil, sunflower oil, and olive oil were procured from local market, and Carbopol-971 was purchased from GLR Innovations India Pvt. Ltd. (New Delhi, India). All other chemicals and solvents used were of analytical grade.

### 4.2. Methods

#### 4.2.1. In-Silico Molecular Docking Study

The crystal structure of Interleukin-6 and TNF-α target proteins was downloaded from a protein data bank. The 3D structure of TQ and FA were retrieved from PubChem databases. The molecular docking tools were MGL, Auto Dock 4.2, and Discovery studio. Auto dock tools were used for finding the Gibbs free energy between ligand and protein during binding. The PDBID of proteins used in this study were 4CNI and 6RMJ. The downloaded protein was prepared for docking by removing water molecules, adding kollman charges (partial charges), adding polar hydrogen atoms, and finally converted into a pdbqt file of our protein. Meanwhile, the active site was identified by a computed atlas of surface topography of protein (CASTp 3.0) [44]. The identification of the binding pocket is the prerequisite for site-specific docking. After the binding pocket analysis, it was suggested that the amino acid, e.g., Pro41, Lys43, Thr93, Leu115, Thr117, Val118, Ser119, Phe153, Glu155, Pro174, Ala175, Leu177, Gly181, Tyr183, Val17, His15, Ala28, Pro20, Ala33, Arg32, Phe144, Asn34, Glu146, Gly148, Ser147, Gln149, Tyr151, and Val150 are present in the active binding pocket of Interleukin-6 and TNF-α protein (Figure 1). For site-specific docking, the generation of a grid file is crucial; therefore, we used grid parameters that contain center_x = 88.031, center_y = −35.585, center_z = 5.813 and size_x = 80, size_y = 72, and size_z = 60 for 4CNI. Similarly, for 6RMJ, center_x = 4.681, center_y = 66.45, center_z = 117.048, size_x = 68, size_y = 58, and size_z = 52. The preparation of ligands is similar to receptor preparation; both ligands were downloaded and converted into a pdbqt file format for docking using Autodock vina work on Lamarkian genetic algorithm that computed the binding free energy for interacting ligands [45]. Discovery studio 2019 was used for visualisation purposes to see the interaction between proteins and ligands.

#### 4.2.2. In Vitro Studies

##### Antioxidant DPPH Free Radical Scavenging Assay

The capacity of TQ, FA, and FTQ-NEG to bleach the DPPH stable radicals was used as a basis to measure their free radical scavenging activity. An amount of 0.5 mL of test/standard sample with a concentration of 10 to 100 μg/mL was prepared and added into 0.5 mL of DPPH. The absorbance of 0.5 mL ethanol and 0.5 mL DPPH was measured spectrophotometrically at 517 nm subsequently with 30 min incubation at a temperature of 37 ± 1 °C in dark. The standard used was ascorbic acid. The fraction of DPPH scavenging was estimated using the same method [46]:

$$\%\;\mathrm{DPPH}\;\mathrm{Scavenging}=\frac{\left[\mathrm{Abs}\;\mathrm{control}-\mathrm{Abs}\;\mathrm{sample}\right]*100}{\mathrm{Abs}\;\mathrm{control}}$$

##### Skin Interaction Study of the Gel Formulation

Fourier transform infrared spectroscopy (FTIR) and Differential scanning calorimeter (DSC) were employed for the investigation of the interaction profile of the formulation FTQ-NEG with mouse skin. Freshly prepared mice skins were placed on two separate Franz diffusion cells, each filled with a phosphate buffer (pH 5.8) in the receptor compartment. NEG was placed in the donor compartment of the Franz diffusion cell and permeation was studied. As a control, skin samples that had not been treated to the NEG setup were placed on another Franz diffusion cell. Skin samples were removed after 24 h and carefully cleaned using distilled water, chopped, and kept for drying at room temperature with the silica gel and sealed in aluminium pans to conduct the DSC temperature range of 30–350 °C. The temperature was increased at a rate of 10 °C/min, and the nitrogen flow was maintained at 60 mL/min. For FTIR Kbr, pellets were made with the skin samples for the analysis. The obtained pellets were then scanned between a 4500–450 cm^−1^ wavenumber in the infrared spectrum of light number of scans (16) and resolution (4 cm^−1^) [47].

##### CLSM Study for Tissue Uptake

The skin samples were placed between the receptor and donor compartments of the Franz diffusion cell for tissue uptake measurement. The donor compartments were filled separately with 0.3% *w*/*v* aqueous dispersion of Rhodamine B, FTQ-NEG, and pure drug TQ solution containing Rhodamine, whereas the receptor compartments were filled with phosphate buffer solution (pH-5.8). After 24 h, samples of the skin were collected and rinsed with a solution of distilled water and alcohol (1:1) for the removal of surplus Rhodamine. Slides were prepared and observed at an excitation wavelength of 540 nm and emission wavelength of 625 nm utilizing an argon laser beam and confocal microscope from Olympus FluoViewTM FV1000, Hamburg, Germany. The penetration depth of aqueous dispersion Rhodamine B, the drug solution, and FTQ-NEG along Rhodamine was compared using the Z-axis [48].

#### 4.2.3. In Vivo Studies

The Central Animal House Facility, Jamia Hamdard, New Delhi, India, provided BALB/c male and female mice weighing between 25 g and 30 g. The study was conducted, after approval of the protocol by IAEC.JH (Approval No. 1794).

##### Skin Irritation Study

BALB/c mice were used for skin irritation test. Hair was removed from the dorsal surface of the mice using Veet hair removal cream. Three groups of animals were taken (each group having 4 animals). Group 1: control (no treatment), Group 2: FTQ-NEG, and Group 3: negative control (standard irritant formalin solution 0.8% *v*/*v*). After 24 h of administering the formulated preparations, mice were checked for signs of edema and erythema. The Draize score test was employed and scores were provided. The score values ranged from 0 to 4, with 0 indicating no changes, 1 suggesting slight markings, 2 indicating moderate changes, 3 indicating marked changes, and 4 indicating extremely noticeable alterations [49]. After visual scoring, the rats were sacrificed and the skin samples were taken for histological assessment.

##### Imiquimod- (IMQ) Induced Psoriatic Plaque-Like Model

BALB/c mice were utilised in the in vivo studies. Test animals were kept at a temperature condition of 25 ± 2 °C, with relative humidity of 45% ± 5% for 12 h of dark and light cycle with no curtailing of food and drink. BALB/c mice were distributed into 6 groups, each with 5 animals. Hair was removed from the dorsal surface of the mice using Veet hair removal cream, and psoriasis was induced by applying Imiquimod cream 5% daily [49,50] (12.5 mg) on each mouse’s shaved left ear and back for eight days. Except for the control normal mice group, details can be found in Table 2. The clinical Psoriasis Area and Severity Index (PASI) was identified as the best grading system based on the severity of inflammation. After eight days of treatment, animals were subject to cervical dislocation, and skin and spleen were removed and kept in a 10% formalin solution. Haematoxylin/eosin dye was used for staining the samples. These samples were compared to a control skin sample under a light microscope (Motic digital microscope, DMB series) [51].

##### Ear Thickness, Body Weight, and Inflammation Measurements via PASI (Psoriasis Area Severity Index) Scoring

The thickness of the ear was measured using a digital calliper and body weight digital balance where the right ear was kept as a control and the amount of inflammation was measured in the left ear. The presence of erythema, scaling, lesions, and thickness on the skin of mouse ear and back skin were used for determining the seriousness of inflammation and redness with a scoring between 0–4, with 0 indicating no change, 1 indicating slight change, 2 indicating moderate change, 3 indicating marked change, and 4 indicating very marked change.

##### Spleen Weight and Histopathology

Inflammatory disorders cause the spleen to expand in size due to increased cytokine production. Spleen enlargement is a key indicator of immunological dysfunction. Splenomegaly can be induced by IMQ due to a raised amount of Th17 cells. The size and form of the spleen was measured in terms of weight and dimension. Spleen samples were fixed in a 10% neutral buffered formalin solution. After staining, the samples were mounted on slides and examined under a microscope [52].

##### Immunohistochemistry Staining (IHC) of TNF-α and IL-6 in Skin and Spleen Tissue

The 3,3-diaminobenzidine (DAB) development method was employed for (IHC) staining. Mouse ICAM-1 monoclonal antibody was used for incubating skin tissues (mAb, Santa Cruz Biotechnology, Inc., Dallas, TX, USA) and the polink-2 with polymerized horse-radish peroxidase (HRP) DAB detection kit was then used to further prepare the samples (Golden Bridge International Inc., Mukilteo, WA, USA) according to protocol set by the manufacturer. An inverted microscope was employed for acquiring the images (Eclipse TS100, Nikon, and Tokyo, Japan) [53].

##### Analysis of Cytokine Levels in Skin Homogenate

In the IMQ-produced psoriatic plaque model, skin homogenates from various treatment groups underwent ELISA tests. The skin tissues were homogenised using an extraction buffer which was made from 10 mM Tris pH 7.4, 150 mM NaCl, and 1% Triton X-100, for 5 min at 3000 rpm using tissue homogenizer (Remi Electrokinetic, Ltd., Vasai East, Thane, Maharashtra, India) followed by centrifugation for 15 min at 10,000 rpm and a temperature of 4 °C. The supernatants were stored at −80 °C until they were analysed. The TNF-α and IL-6 concentrations were analysed by utilizing the appropriate kits as per manufacturer’s recommendations using quantitative kits of Mouse ELISA (Krishgen biosystem) [14].

##### Statistical Analysis

GraphPad Prism 6.0 was used for the statistical analysis (version 6.05, GraphPad Software, Inc., Boston, MA, USA). A two-way and one-way ANOVA was applied on the collected data, after which Bonferroni’s multiple comparisons test was employed, with a statistical significance of *p* < 0.05.

## Figures and Tables

**Table 1:** Docking score, hydrogen, and hydrophobic interaction of ligands with target receptors.

Protein (PDB:ID)	Compound Name (PubChem ID)	Docking Score (kcal/mol.)	Hydrogen Bonding Interactions	Distance of Hydrogen Bond (Å)	Hydrophobic Interactions
IL-6 (4CNI)	TQ(10281)	−4.4	Leu11 N…O2	3.12	Pro154 (alkyl), Pro156 and Pro209 (pi–alkyl)
	FA(5359407)	−6.0	Val118 O…H24 Thr93 OG1…O23 Ala175 O…H26 Tyr183 OH…O2	2.93 2.91 2.12 3.08	Pro41 and Leu177 (pi–alkyl)
TNF-α (6RMJ)	TQ(10281)	−4.8	Ala145 NH…O2 Phe144 NH…O2	2.67 2.22	Phe144 (alkyl, pi–pi, T-shaped), Leu142 (pi–alkyl)
	FA(5359407)	−6.4	Asp143 OD1…H20 Asp143 HA…O19 Phe144 NH…O19 Asp140 HA…O25	2.41 2.84 2.14 3.05	Phe144 (pi–pi, T-shaped)

**Table 2:** Groups of animals (n = 5).

S. No	Groups	Category	No. of Mice	Duration/Days
1	Group-1	Control group (No treatment)	5	8
2	Group-2	Negative control (Disease induced)	5	8
3	Group-3	Positive control (Tenovate M cream; 40 mg/cm^2^ marketed formulation)	5	8
4	Group-4	Free drug (FD) TQ in gel (50 mg/cm^2^)	5	8
5	Group-5	FTQ-NEG (50 mg/cm^2^)	5	8
6	Group-6	FA in gel (100 mg/cm^2^)	5	8

## Data Availability

Not applicable.
